# Environmental DNA analysis as a non‐invasive quantitative tool for reproductive migration of a threatened endemic fish in rivers

**DOI:** 10.1002/ece3.4653

**Published:** 2018-11-06

**Authors:** Atsushi Maruyama, Kousuke Sugatani, Kazuki Watanabe, Hiroki Yamanaka, Akio Imamura

**Affiliations:** ^1^ Faculty of Science and Technology Ryukoku University Otsu Shiga Japan; ^2^ Hokkaido University of Education Asahikawa Hokkaido Japan

**Keywords:** endangered fish, Lake Biwa, quantitative PCR, seasonal migration, spawning, species‐specific eDNA

## Abstract

Quantitative information regarding reproduction is essential for conserving endangered animals; however, some conventional quantitative methods can be damaging to the target population and their habitats. In the present study, the reproductive migration of a threatened endemic fish, three‐lips (*Opsariichthys uncirostris uncirostris*), was non‐invasively monitored by quantitative PCR of species‐specific environmental DNA (eDNA), the usefulness of which has been not sufficiently explored. Water sampling and from‐shore visual inspection were performed weekly along a tributary of Lake Biwa (Japan), where adult fish seasonally migrate upstream to reproduce as well as at lake sites near the river mouth. Species‐specific eDNA was collected at all locations at times when the fish were visually observed and at certain sites where the fish were not observed. Log‐transformed individual counts from visual inspection were positively correlated with log‐transformed eDNA concentration in the river sites, indicating that eDNA analysis can be a reliable quantitative tool for fish abundance in rivers. Furthermore, distance from the lake did not influence eDNA concentration, suggesting that eDNA transport by river flow had a negligible effect on eDNA quantification. Both eDNA concentration and individual counts gradually increased from May–July, and decreased in August. Importantly, eDNA analysis showed that the fish occupied more habitats in the peak reproductive season and stayed for longer time at any given site. An additional underwater survey confirmed unexpected eDNA detections as true positives. eDNA analysis has great potential to quantitatively monitor reproductive fish migrations under certain conditions.

## 1 INTRODUCTION

Information regarding reproduction is essential for effectively and efficiently conserving rare and endangered animal species (Sutherland, 1998). However, since they often involve capturing animals and/or invading breeding grounds for direct observation during breeding seasons, conventional methods of surveying reproductive ecology are potentially damaging to the target species and its habitat. In addition, conventional survey methods require experts to be present for capture and/or species identification; thus, high financial costs can prevent the long‐term monitoring of target species. Therefore, non‐invasive, accessible approaches for studying reproductive ecology are required. Recently, environmental DNA (eDNA) analysis was proposed as a non‐invasive, quick approach to monitoring aquatic macro‐organisms (Ficetola, Miaud, Pompanon, & Taberlet, 2008; Jerde, Mahon, Chadderton, & Lodge, 2011; Lodge et al., 2012). This technique can reveal the distribution of target species by detecting species‐specific DNA fragments in water samples. eDNA analysis can be performed non‐invasively and simultaneously at several sites with more repetition than conventional methods, such as capture and direct observation, because the only fieldwork necessary is the collection of water samples (Darling & Mahon, 2011; Nakagawa et al., 2018; Yamamoto et al., 2016).

In addition to the detection of the target species presence, estimates of species abundance are also essential for conservation, for example, detecting drastic decreases in abundance to warn of possible extinctions. eDNA analysis has the potential to estimate the abundance or biomass of aquatic animals; under controlled conditions in experimental aquariums and mesocosms, the amount of DNA released by organisms is related to their abundance or biomass (Klymus, Richter, Chapman, & Paukert, 2015; Maruyama, Nakamura, Yamanaka, Kondoh, & Minamoto, 2014; Mizumoto, Urabe, Kanbe, Fukushima, & Araki, 2017; Takahara, Minamoto, Yamanaka, Doi, & Kawabata, 2012). Despite this, degree of correlation between eDNA concentration and abundance demonstrated in the field is variable between studies. A recent study of a stream‐dwelling char demonstrated a high correlation (Wilcox et al., 2016), but other studies reported marginally significant correlations or secondary contribution of abundance to eDNA amount (Doi et al., 2016; Erickson et al., 2016; Lacoursière‐Roussel, Côté, Leclerc, & Bernatchez, 2016; Klobucar, Rodgers, & Budy, 2017; Nevers et al., 2018; Pilliod, Goldberg, Arkle, Waits, & Richardson, 2013; Thomsen et al., 2012; Yamamoto et al., 2016). In some studies, eDNA concentration did not correlate with the biomass of the target animals (Biggs et al., 2015; Spear, Groves, Williams, & Waits, 2015). Poor correlation, which can compromise the reliability of eDNA analysis for estimating animal abundance, may be explained by overdispersion of data generated by both eDNA analysis and conventional methods. Chambert, Pilliod, Goldbeerg, Doi, and Takahara (2018) listed several factors that result in the overdispersion of eDNA data, including variation in individual shedding rates (Klymus et al., 2015; Maruyama et al., 2014; Mizumoto et al., 2017), uneven distribution of animals in the environment (Lacoursière‐Roussel et al., 2016; Laramie, Pilliod, & Goldberg, 2014; Pilliod et al., 2013), environmental disturbance (Barnes & Turner, 2016) and sampling methods and environmental conditions (Goldberg, Pilliod, Arkle, & Waits, 2011; Lacoursière‐Roussel et al., 2016; Pilliod, Goldberg, Arkle, & Waits, 2014).

**Figure 1:**
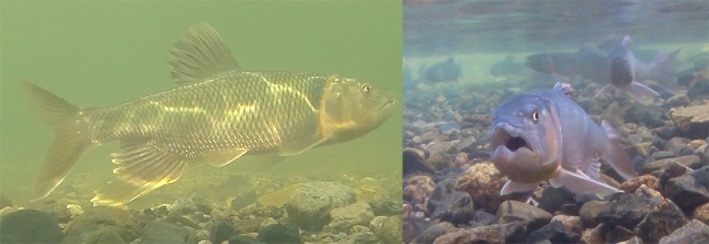
Underwater photographs of a threatened endemic fish, three‐lips (*Opsariichthys uncirostris uncirostris*), in a tributary of Lake Biwa, Japan

To assess whether animal density can be predicted by eDNA analysis, we selected a cyprinid fish, three‐lips (*Opsariichthys uncirostris uncirostris*, Temminck *et* Schlegel), in a river as a model to test whether eDNA analysis can quantitatively monitor fish populations (Figure 1). This fish grows in lakes and large adult individuals (14–25 cm standard length; 2^+^–4^+^ age) only seasonally migrate upstream into tributaries for reproduction (Nakamura, 1951; Tanaka, 1970). Therefore, the body size of individuals in rivers is similar, which minimizes any possible effect that size may have on eDNA shedding rate (Maruyama et al., 2014). In addition, large three‐lips individuals are easy to count from land (Imamura, 2014), enabling accurate abundance estimation by visual survey. In addition, a quantitative monitoring technique is required for the conservation of this unique piscivorous Japanese cyprinid subspecies, endemic to lakes Mikata and Biwa. The native populations are listed as vulnerable in the Red List specific to Japan (Japan Ministry of the Environment, 2010), because the Lake Mikata population is considered extinct, and the catch in Lake Biwa has been decreasing continuously since the 1980s (Tsunoda, Urano, & Ohira, 2015). In spite of this, the patterns of reproduction or migration of this species have not been studied since Tanaka (1970) investigated the age structure and growth rate of its spawning populations in the 1960s.

The objective of this study was to clarify the pattern of three‐lips reproductive migration along a tributary of Lake Biwa by monitoring change in species abundance estimated by eDNA analysis as well as riverside and lakeside visual surveys. eDNA was validated as a method for quantitative fish monitoring in rivers. Environmental factors affecting estimates of fish abundance by eDNA analysis were also explored.

## 2 MATERIALS AND METHODS

### 2.1 Study sites

**Figure 2:**
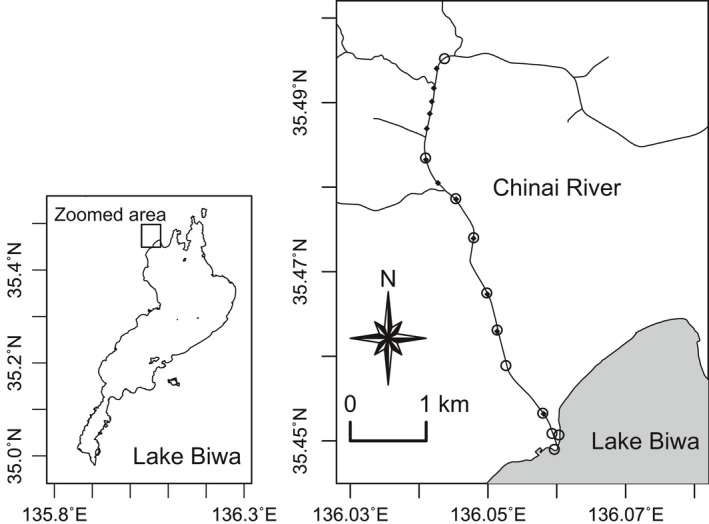
Locations of the survey sites (open circles) along the Chinai River (*n* = 9) and Lake Biwa shore (*n* = 2) in central Japan. Solid diamonds indicate positions of a river‐crossing fish trap (*n* = 1, the closest from the lake) and small dams (*n* = 11). All the survey sites located near the trap or dams (*n* = 6) were settled 3 m upstream from the trap or dams

We selected the Chinai River, a tributary of Lake Biwa in central Japan, as a model site for study (Figure 2) because three‐lips was reported to migrate along this river in greater numbers than along the other tributaries of Lake Biwa when the fish was still abundant (Nakamura, 1951). According to our 2016 preliminary survey, during its reproductive season, the fish is now most abundant in this river among the 12 largest tributary rivers on the west side of Lake Biwa, probably because unlike most other tributary rivers that dry up seasonally in the summer, this river maintains surface water all year‐round. There is a fish trap spanning the width of the watercourse, and there are 11 small dams (0.22–1.17 m high) on the Chinai River, some of which might be barriers to the migration of fish.

Nine study sites along the Chinai River (180–5,800 m from the river mouth) and two sites on the lakeshore (within 100 m to the north and south of the river mouth) were selected (Figure 2). All the study sites near the trap or dams (*n* = 6) were at least 3 m upstream from the trap or dams. The other river sites (*n* = 3) were 3 m upstream of the borders of different types of riverbed substrate (sand or gravel). The width and the depth of the river were 5–20 m and 15–50 cm, respectively, in the study area.

The field survey was conducted weekly from 10th May to 21st September 2017 (20 weeks) to cover the three‐lips reproductive migration season (Nakamura, 1951). All the 11 sites were visited in the day time within one day once per week for water sampling, visual inspection and other measurements described below. Due to flooding, the field surveying was canceled four times. Thus, each site was visited 16 times in total, while some samples in the upper reaches of the river were not analyzed in off‐peak seasons to reduce costs (see Section 2.1).

### 2.2 Quantitative environmental DNA analysis

For eDNA analysis, 500 ml water was collected using a polypropylene bottle at each site and filtered on site using a glass fiber filter (Whatman GF/F, 0.7 µm pore size, GE Healthcare, Chicago, US) and a plastic filter funnel (ASONE, Osaka, Japan) within 90 min of sampling to ensure that eDNA decay was less than 10% (Barnes et al., 2014; Maruyama et al., 2014). To provide negative controls, distilled water was filtered at the beginning and end of each day. Immediately after each sample was filtered, 70% ethanol was added to the samples for eDNA preservation and the samples were preserved in portable freezers (Minamoto, Naka, Moji, & Maruyama, 2016). Before use, all the sampling and filtering equipment was washed with a 10% bleach solution to remove any residual DNA fragments and rinsed with distilled water to remove residual bleach. The sampling bottles were not reused over the course of each day.

DNA was extracted from 185 samples (including 32 negative controls) using a DNeasy Blood & Tissue Kit (Qiagen, Hilden, Germany) in combination with a spin column (EP‐11201, Gene Design, Ibaraki, Japan) following the method suggested by Miya et al. (2015) with slight modifications. The filter was tightly folded into a small cylindrical shape and placed into a spin column from which the attached silica gel membrane had been removed in advance. The spin columns were centrifuged at 6,000 *g* for 1 min to remove excess ethanol from the sample. 100 µl buffer AL and 10 µl proteinase K were mixed with 200 µl Milli‐Q water to increase the volume enough to penetrate all the filter. The mixture was dispensed onto the filter in each spin column, and the spin columns were incubated for 30 min at 56°C. After incubation, the spin columns were centrifuged at 6,000 *g* for 1 min to collect DNA and the eluted filtrate was transferred to a new 2.0 ml collection tube. To recover residual DNA on the filter, 200 µl TE (Tris‐EDTA) buffer was added to each filter and the filter was incubated for 1 min at room temperature before being centrifuged at 6,000 *g* for 1 min. The upper part of the spin column containing the filter was removed from the 2.0 ml collection tube, and the first and second filtrates were combined in the 2.0 ml collection tube. Then, 100 µl buffer AL and 600 µl ethanol were added to the combined filtrates and mixed well by gently pipetting. The DNA in each mixture was purified with the DNeasy Blood & Tissue Kit as per the manufacturer's instructions. Each mixture was transferred to a new spin column from the DNeasy Blood & Tissue Kit to trap DNA fragments on the silica gel membrane by centrifugation. Because of the large volume of each mixture, this step was repeated twice to ensure that all DNA was caught on the membrane. Then, the silica gel membrane was washed twice with the washing buffers AW1 and AW2, and DNA was eluted from the column with 100 µl buffer AE. All the DNA extracts were frozen at −20°C until PCR amplification.

The quantification of the eDNA concentration was performed by real‐time quantitative TaqMan^®^ PCR with a Real‐Time PCR system (Applied Biosystems^®^ StepOnePlus^TM^, Thermo Fisher Scientific, Waltham, USA). The mitochondrial D‐loop gene 129‐bp fragments were amplified and were quantified with forward and reverse primers, Oun_Dlp_F (5′‐CATTTCCTTGCCAGGCTTAATAATA‐3′) and Oun_Dlp_R (5′‐GCAAAAGGGGGCATATATATAAGAGA‐3′), respectively, and a probe, Oun_Dlp_Pr (5′‐FAM‐**C**ATAT**G**TTTAT**C**T**C**AT**G**T**GC**ATAA**C**‐TAMRA‐3′), where bold **C** and **G** indicate locked nucleic acids (LNAs) that increase the melting temperature. Specificity of the primer–probe set had been confirmed previously by PCR with tissue samples of the three fishes most closely related to three‐lips dwelling in the same region, namely *Zacco platypus*,* Nipponocypris temminckii,* and *N. sieboldoii* (Yamanaka, Takao, Maruyama, & Imamura, 2018). Each TaqMan® reaction contained 900 nM of each primer and 125 nM TaqMan® probe in the PCR master mix (TaqMan® Environmental Master Mix 2.0, Thermo Fisher Scientific) as well as 0.075 µl AmpErase®Uracil N‐Glycoslase (Thermo Fisher Scientific) and 2 µl of the DNA template. The total volume of each reaction mixture was 15 µl. The PCR conditions were as follows: 2 min at 50°C and 10 min at 95°C, followed by 55 cycles of 15 s at 95°C and 60 s at 60°C. The quantitative real‐time PCR (qPCR) was performed in triplicate for each eDNA sample. To quantify the number of three‐lips D‐loop genes in each 2 μl eDNA template, we performed qPCR in triplicate simultaneously using a dilution series of standards containing 60, 600, 6,000, and 60,000 copies of the target sequences as standards in all qPCR assays. The standards were provided by a commercial service (Standard Genes, Eurofins Genomics K.K., Tokyo, Japan) in which the target sequence was cloned using pEX‐K4J1 vector. The product was delivered as plasmids and used as the source of the dilution series. The solution of standard was stocked in a −20°C freezer at the concentration of 300,000 copies/µl, and the dilution series were prepared freshly for each PCR experiment. The *R* ^2^ values of the standard regressions ranged from 0.987 to 0.996, though PCR efficiencies were low (67.2%–82.4%). The low efficiency of PCR may cause false negatives; however, the estimated eDNA concentrations in the sample waters seem to be reliable as they correctly reflect the copy numbers of target DNA in PCR templates with preserving intercorrelation of eDNA concentrations among samples. Three wells of negative control samples (containing no template) were included in all the qPCR assays. The samples for which qPCR triplicates showed no amplification (Ct > 55) were considered negative for three‐lips eDNA. The concentration of DNA (copies/L) in sample water was calculated based on the mean quantity of qPCR triplicates in each water sample, where negative replications were included as zero, and used in statistical analyses as eDNA concentration. Instead of setting arbitrary limits of detection and quantification, we statistically analyzed all positive concentration data Section 2.4).

To confirm that the qPCR amplicons were of the target species, the sequences of qPCR amplicons were determined using a commercial sequencing service (Takara Bio, Kusatsu, Japan) after treatment with ExoSAP‐IT (USB Corporation, Cleveland, OH, USA). Out of 185 qPCR amplicons, the 33 samples containing the least DNA (Ct ≥ 40) were selected for sequencing with the forward and reverse primer Oun_Dlp_F. The 27 samples with the second least DNA were selected for sequencing only with the reverse primer Oun_Dlp_R.

### 2.3 Visual inspection and measurements of environmental conditions

Immediately after water sampling at each site, the number of individual three‐lips within a 30 m stretch upstream of the water sampling site was determined by visual inspection from the riverbank, bridge, or lakeshore. The same researcher (KS) performed the visual inspections throughout the study. The observer went into the water only when conclusive species identification was not possible from the shore. When three‐lips were found in groups, they were observed carefully for 10 min to determine whether they were spawning.

Afterward, we measured the pH of the water using a compact pH meter B‐712 (Horiba Ltd., Kyoto, Japan), conductivity using a compact meter B‐771 (Horiba Ltd.), water temperature using a digital meter ND‐X (Marukan Ltd., Osaka, Japan), current velocity using a propeller current meter CR‐11 (Cosmo Riken Ltd., Osaka, Japan), and coordinates (latitude and longitude) using an iPhone 7 (Apple Inc., Cal., US) with a Google Maps application (Google Inc., Cal., USA). The type of riverbed substrate over each 30 m observation site was classified by eye as gravel or sand during the first survey on 10th May 2017. No other types of substrate were observed within the study sites. In the two lake sites, lake bottom was sandy as far as the eye could see.

At the beginning of the apparent peak of the reproductive season, an additional underwater survey was conducted by drift snorkeling. The underwater survey was done on the next day of the 10th survey (i.e., 13th July 2017) to avoid possible cross‐contamination through the snorkeling equipment and disturbance to the fish. An experienced observer (AM) swam down the river from the uppermost site (5,800 m from the lake) to the fifth‐highest site (2,350 m from the lake), where three‐lips had not been observed during visual inspections. The observer was able to identify any individual fish larger than ~10 cm standard length in the whole area between the both shores. Underwater survey was not appropriate for counting the three‐lips, which tended to flee from observers in the water; the underwater survey was conducted only to investigate possible false positive eDNA analysis results in the upper reaches of the river where the fish were not observed from the riverbank or bridge.

### 2.4 Data analyses

Since measuring the distribution and abundance by quantitative eDNA analysis and visual inspection usually gives zero‐inflated data (negative results), the data were compared by statistical analysis in four steps as follows. First, using all the 153 data points, the relationship between the presence (positive/negative) of three‐lips according to eDNA analysis and according to visual inspection was examined using Fisher's exact test. Second, using 89 positive eDNA data points, the effect of normalized (log_10_‐transformed) eDNA concentration and differences between habitats (river vs. lake) on the probability of positive detection by visual inspection was examined using a generalized linear model (GLM) with a logit link function. The interaction term of the eDNA concentration and habitats was excluded from the model because of the large error of the estimate of its effect (*P* ≈ 0.8). Third, using 32 positive visual inspection data points, a linear model (LM) was calculated to predict the individual number of three‐lips observed during visual inspection based on the log_10_‐transformed eDNA concentration. This was done for river and lake sites separately. Finally, the factors affecting residuals of a significant LM for river sites (see Section 3) were analyzed using another LM (residual analysis) with stepwise (forward and reverse) variable selection based on Akaike's information criteria (AIC) from six potential environmental factors, namely distance from the lake, pH, conductivity, water temperature, current velocity, and riverbed substrate, as explanatory variables. To examine the effect of eDNA flow from the upper reaches of the river, the distance from the lake was included; if the effect was not negligible, a positive effect was expected on the residuals of visual counts. pH and conductivity can affect eDNA collection efficiency (Tsuji, Yamanaka, & Minamoto, 2017). Temperature and current velocity may affect the rates of eDNA release and decay (Pilliod et al., 2014; Strickler, Fremier, & Goldberg, 2015; Tsuji, Ushio, Sakurai, Minamoto, & Yamanaka, 2017). Substrate type may affect DNA sedimentation and detection efficiency by visual inspection.

The seasonal changes in distribution and abundance of three‐lips were examined in two steps. First, using detection data (positive/negative) from the river, the relationship between the number of dates on which fish were detected and distance from the lake at each river site was examined separately for the two methods using Spearman's rank correlation test. Second, using quantitative data from the river, changes in estimates of the fish quantity (eDNA concentration and visual counts) were examined, before and after the first observation of spawning behaviors and separately for the two methods, by LM with time (weeks after the first survey), distance from the lake (m) and an interaction term for these two continuous variables as explanatory variables. Estimates of three‐lips abundance in the lake were examined by LM in the same manner, but with time as the only explanatory variable.

Fisher's exact test, GLM, LM, and stepwise variable selection were conducted using “fisher.exact,” “glm,” “lm” and “stepAIC” functions, respectively, in R ver. 3.1.2 (R Core Team, 2015). The significance level was set at 0.05 in all analyses.

## 3 RESULTS

### 3.1 Validity of eDNA analysis in comparison to visual inspection

There was no amplification of the three‐lips species‐specific eDNA in any distilled water samples filtrated before and after test water samples or in any negative control using qPCR. Thus, inter‐sample contamination was considered to be negligible throughout the analysis. DNA sequencing after qPCR confirmed that the sequences of all 93 amplicons examined were from three‐lips.

eDNA analysis detected three‐lips eDNA in 89 out of 153 samples, whereas the fish were only visually observed 32 times during 153 inspections (Table 1). Importantly, eDNA was detected in all water samples from sites where three‐lips were observed by visual inspection. The number of positive eDNA analysis results was significantly larger than the number of positive visual inspection results (Fisher's exact test, *p* < 0.001).

**Table 1:** Relationship between the detection of three‐lips by the environmental DNA (eDNA) analysis and visual inspection, which were concurrently conducted 16 times during May–September 2017 at nine river sites and two lake sites

	eDNA analysis	
	Positive	Negative
Visual inspection		
Positive	32	0
Negative	57	64

Note

Numbers of data sets, each of which is derived from an eDNA sample and an inspection at a site on a day, are shown.

**Figure 3:**
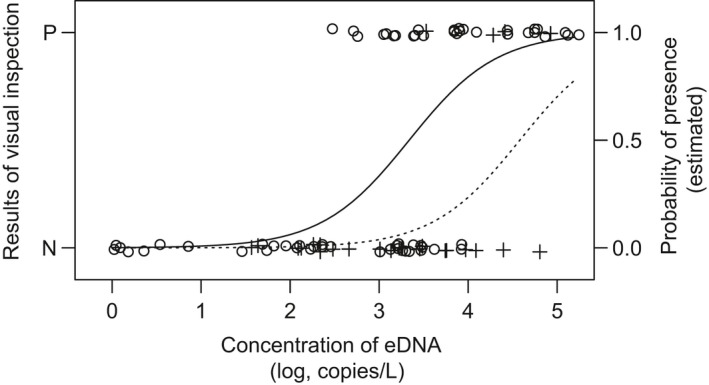
The results of visual inspection (P: positive, N: negative) of three‐lips (left Y‐axis, plots) as a function of its species‐specific environmental DNA (eDNA) concentration (log_10_‐transformed). Open circles and crosses indicate river and lake survey sites, respectively. These plots are vertically staggered to minimize overlapping. 89 positive eDNA data points were used (Table 1). The solid and dotted lines (right Y‐axis) indicate probabilities of detection by visual inspection estimated by logistic models for river and lake survey sites, respectively

When the 89 positive eDNA data points were analyzed, it emerged that the probability of observing three‐lips during visual inspection was significantly correlated with the log_10_‐transformed eDNA concentration (GLM with a logit link function, *z* = 4.12, *p* < 0.001; Figure 3) and was significantly different between the river and lake sites (*z* = 3.18, *p* < 0.01).

**Figure 4:**
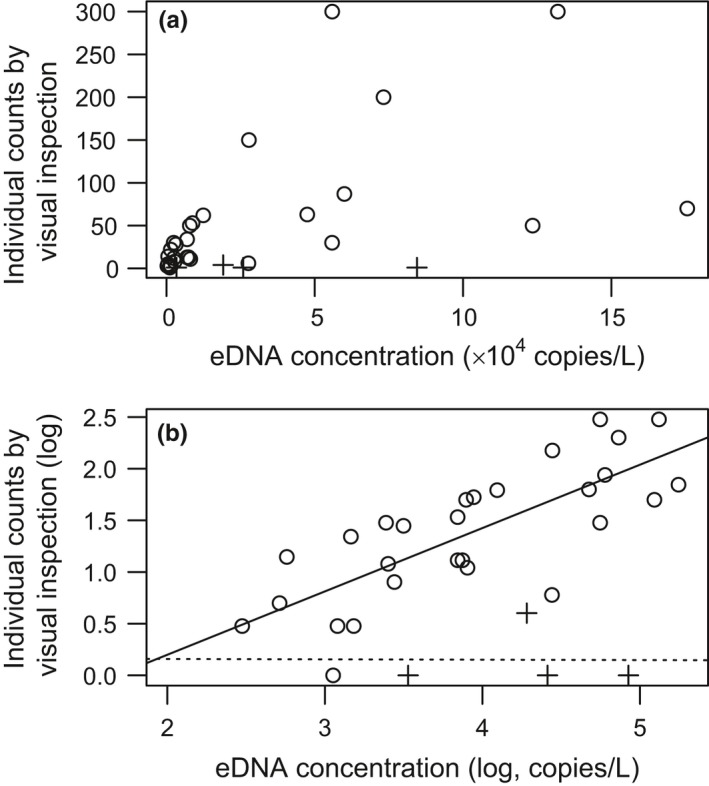
Scattered plot (a) and log_10_–log_10_ plot (b) of individual counts per 30 m of three‐lips by visual inspection and its species‐specific environmental DNA (eDNA) concentration. Open circles and crosses indicate river and lake survey sites, respectively. 32 positive visual inspection data points were used (Table 1). The solid and dotted lines indicate linear models for river and lake sites, respectively

When the 32 data points positive in both analyses were analyzed, the log_10_‐transformed individual number of three‐lips observed by visual inspection was positively correlated with the log_10_‐transformed eDNA concentration at the river sites (LM, *R* ^2^ = 0.574, *p* < 0.001; Figure 4), but not at the lake sites (*R* ^2^ < 0.1, *p* > 0.5). In the river, the individual counts from visual inspection (*N*) can be predicted from eDNA concentration (*C*) as log_10_ *N* =–0.842 + 0.6131 × log_10_ *C*.

**Figure 5:**
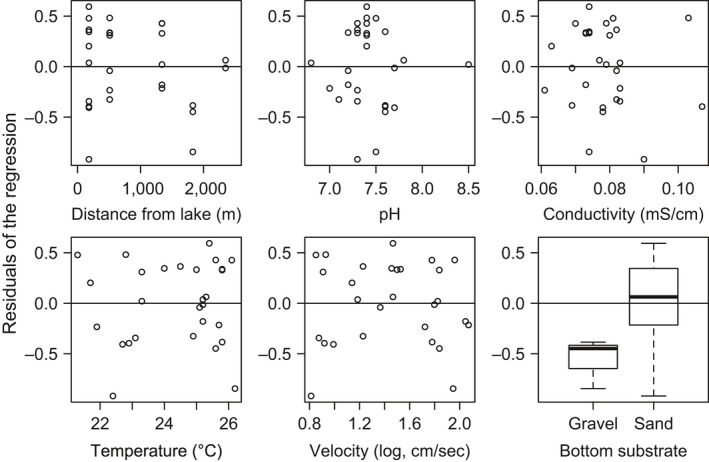
The residuals of the linear model (on visual counts by eDNA concentration) for river survey sites (solid line in Figure 4b) as functions of environmental factors: distance from the lake, pH, conductivity, water temperature, log_10_‐transformed current velocity, and riverbed substrate. Horizontal solid lines indicate residual = 0. In the box plot, bold lines, boxes, and bars indicate medians, quartile ranges, and maximum–minimum ranges, respectively

Water temperature and riverbed substrate were selected as explanatory variables by stepwise variable selection using AIC. The residuals of the regression (Figure 4) were significantly explained by the riverbed substrate (LM, *t* = 3.16, *p* < 0.01; Figure 5), but not by water temperature (*t* = 1.37, *p* = 0.182). The residual was larger at the sites with sand substrate than at the sites with a gravel substrate. In other words, more three‐lips individuals were counted than expected based on the eDNA concentration at the sites with sand substrate than at the sites with gravel substrate.

### 3.2 Seasonal changes in distribution and abundance monitored by eDNA analysis and visual inspection

**Figure 6:**
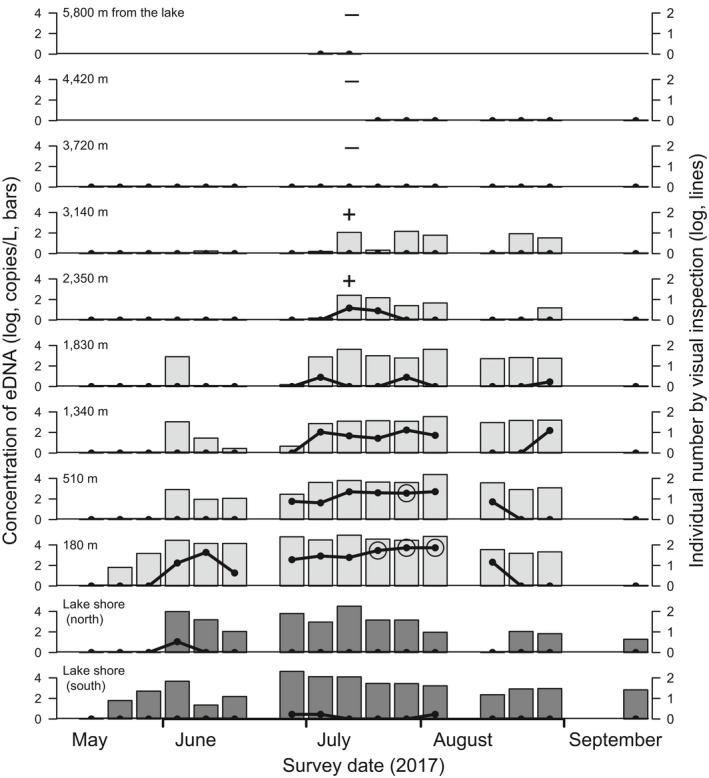
The weekly data of three‐lips species‐specific environmental DNA (eDNA) concentration (log_10_‐transformed; left *Y*‐axis, bars) and individual counts per 30 m by visual inspection (log_10_‐transformed; right *Y*‐axis, plots and lines) at nine river sites (light gray bars, 180–5,800 m upstream from the lake) and two lake sites (dark gray bars). Open circles on the plots indicate observation of spawning behaviors. Spaces without plots indicate skipped surveys due to heavy rain (3 days) or to save cost (others). “+” and “–” indicate positive and negative detection of the fish by underwater survey, respectively

The detection data from the eDNA analysis and visual inspection along the river indicated that the target fish expanded its distribution upstream from the river mouth for 1–2 months (Figure 6). The number of dates on which fish were detected decreased with site distance from the lake, both for eDNA analysis (*ρ *= –0.962, *p < *0.001) and visual inspection (*ρ* = –0.957, *p < *0.001). eDNA analysis and visual inspection recorded fish up to 3,140 and 2,350 m from the lake, respectively. Interestingly, in all cases, the fish presence at each site was detected by eDNA analysis before visual inspection across the entire survey period. During the additional underwater survey from the uppermost site (5,800 m from the lake), fish were first detected at 3,160 m from the lake. At the two study sites in the lake, the target fish was detected by both methods; however, it was observed four times only over 32 visits by visual inspection.

Spawning behaviors were first observed by eye on 19th July in the lower reaches of the river and continued for three weeks (Figure 6). The quantitative data from the two methods in the river showed significant increases in abundance with time, until the beginning of spawning behaviors (Table 2). The slope of the increase in eDNA concentration significantly decreased with distance from the lake, as shown by the interaction term (distance × time), and hence, the peak tended to be higher in the lower reaches of the river than in the upper reaches. After the first observation of spawning behaviors, both the eDNA concentration and individual counts significantly decreased with time. The slope of this decrease was steeper at the sites closer to the lake.

**Table 2:** Coefficients (±standard errors) of linear models (LM) to explain the changes in three‐lips species‐specific environmental DNA (eDNA) concentration and individual counts of the fish by visual inspection (visual)

	Before beginning of spawning				After beginning of spawning			
	eDNA		Visual		eDNA		Visual	
River									
Time (week)		6,000 ± 1,700**		4.7 ± 1.5**		–5,500 ± 1,500***		–18 ± 4***	
Distance (m)		2.1 ± 4.9		0.0005 ± 0.0044		–32 ± 9***		–0.10 ± 0.02***	
Time × distance		–1.6 ± 0.7*		–0.0011 ± 0.0006		1.7 ± 0.6**		0.0056 ± 0.0016**	
Lake									
Time (week)		4,600 ± 1,600*		0.006 ± 0.079		–430 ± 140*		–0.016 ± 0.025	

Notes

LM were separately conducted for the two periods of time (before and after the first observation of spawning behaviors), the two methods (eDNA and visual), and river and lake sites.

Significance level: *p < 0.05; **<0.01; ***<0.001.

In contrast, the results of the two methods did not match in the lake. The eDNA concentration significantly increased with time until the beginning of spawning behaviors, but individual counts did not (Figure 6; Table 2). After the first observation of spawning behaviors, neither the eDNA concentration nor the individual counts changed with time. Consequently, eDNA was collected until the end of the survey period in the lake sites only.

## 4 DISCUSSION

### 4.1 eDNA concentration as a quantitative indicator of fish abundance in rivers

eDNA analysis detected three‐lips eDNA at all the locations and times when the three‐lips was visually observed, without any false negative results. This result strongly supports the detection power of eDNA analysis with species‐specific primers in the field that many previous studies have suggested (Jerde et al., 2011; Lodge et al., 2012; Yamanaka & Minamoto, 2016). Target eDNA was also detected in many samples taken when and where the fish was not visually observed. This does not necessarily imply that eDNA analysis is a more powerful detection tool than visual inspection; false positive results can also explain this situation (Yamamoto et al., 2016). Thus, we checked the sequence of qPCR amplicons and possible inter‐sample contamination. In addition, we performed an underwater survey at the apparent peak of the reproductive season and found that the positive eDNA detection at the uppermost site was not a false positive result. Furthermore, a GLM with a logit function (regression analysis) showed that the probability of visual detection was not independent of the eDNA concentration. All these results suggest that eDNA accurately detected presence of the fish. In addition, the regression analysis showed a higher probability of visual detection in the river than in the lake by one order in eDNA concentration. Since the depth gradient makes visual detection difficult in the lake, and unpredictable water current and eDNA diffusion may have made eDNA analysis inaccurate in the lake (Dunker et al., 2016; Eichmiller, Bajer, & Sorensen, 2014; Ghosal, Eichmiller, Witthuhn, & Sorensen, 2018), this difference is not surprising. Researchers should pay attention to the unreliability of conventional data as well as to that of eDNA data (Chambert et al., 2018). It is also notable that both positive and negative visual detection results were obtained in the range of eDNA concentration widely from 10^2^ to 10^3.7^ copies/L (see Figure 3), probably because of an uneven detection efficiency of both methods, depending on the environmental heterogeneity within the river.

The scatter plot of individual counts by visual inspection and eDNA concentration (Figure 4a) suggests that the estimation of fish abundance is difficult or impossible, even under optimal conditions such as in our study, where large adult individuals (14–25 cm in standard length; 2^+^–4^+^ age) of the target fish only seasonally migrate upstream into tributaries for reproduction (Nakamura, 1951; Tanaka, 1970). However, the log–log plot and the associated statistical analyses strongly support the possibility of accurate abundance estimation in the river, which implies that eDNA quantification can detect the order of magnitude variations in fish abundance under desirable conditions. Thus, quantitative eDNA analysis can be a good indicator of animal density, provided order‐level resolution of quantification is understood, as the present study shows. Importantly, the residual analyses also indicated that most environmental conditions examined do not affect abundance estimations at the log scale, although some of these factors (i.e., pH, conductivity, and temperature) may have potential to affect abundance estimation when their ranges are wider than in our study. Notably, the distance from the lake did not affect the estimation. This result suggests that eDNA transport from the upper reaches of the river has a negligible effect on quantitative analysis at the spatial scale of this study; significant eDNA transport would have yielded a negative relationship between distance and eDNA concentration as long as eDNA concentration is considerably high in the upper reaches (Jane et al., 2015; Rice, Larson, & Taylor, 2018). However, a water current of 10–10^2^ cm/s (Figure 5) carries eDNA roughly 360–3,600 m downstream per hour, during which time only 5%–15% eDNA decays under experimental conditions (Klymus et al., 2015; Maruyama et al., 2014). These facts suggest that some mechanisms other than DNA decay, such as sedimentation and substrate trapping, may decrease the amount of eDNA detectable in the field (Shogren et al., 2016, 2017 ). Further studies are required to understand the quantitative effect and mechanisms of eDNA flow in lotic water (Seymour et al., 2018); however, our findings would support the use of quantitative eDNA analysis in lotic environments. In contrast, the use of eDNA for the quantification in the lake was not validated in this study, because the depth gradient makes counting fish by eye difficult and also because unpredictable water currents and eDNA diffusion may have made the eDNA analysis inaccurate, as stated above (Dunker et al., 2016; Eichmiller et al., 2014; Ghosal et al., 2018).

### 4.2 Seasonal distribution and abundance of three‐lips

Seasonal migration of three‐lips into rivers had not been studied since Tanaka (1970) reported the age structure and growth rate of its spawning populations. Thus, we had no a priori knowledge of seasonality in upstream migration or distribution of three‐lips in its peak breeding season. This study used eDNA analysis and visual inspection to show that the target fish expands its distribution upstream from the lake gradually, and remained in the river for 3–4 months. An important difference between the results of the two methods was eDNA analysis showed a larger distribution at the peak of the reproductive season as well as longer residence time at any given site. Such novel knowledge provided by eDNA analysis may have important implications for the conservation of rare and endangered animal species, for example, when authorizing appropriate fishing areas and seasons.

The quantitative data from both methods in the river showed a gradual increase in abundance followed by a decrease at each site, which indicated the peak season of the three‐lips reproductive migration clearly. In addition, it was shown that more individuals use the lower reaches of the river than the upper reaches. The eDNA concentration in the lake suggests that the fish gather at the river mouth from May until July. Afterward, unlike at river sites, the eDNA concentration remained high, which suggests the fish remains near the river mouth after reproduction. This is also novel information provided by eDNA analysis that may have conservational implications. We propose that several other fish species with similar life histories living in Lake Biwa and its tributaries could be quantitatively monitored using eDNA analysis. This would result in further novel findings obtained via non‐invasive methods.

## 5 CONCLUSION

In the present study, we monitored the reproductive migration of a threatened endemic fish, three‐lips, by quantitative eDNA analysis accompanied by visual inspection. Species detection by eDNA analysis was shown to be accurate and demonstrated a larger distribution and longer residence time of the target fish in the river. In addition, we showed that eDNA analysis, usefulness of which has been considered limited in this context previously, can be a good quantitative tool for fish in rivers at the log scale. Estimating fish abundance using eDNA concentration demonstrated a gradual increase and decrease in the number of fish present. Thus, we propose that, under certain conditions, eDNA analysis has greater potential than conventional methods for quantitatively monitoring the reproductive migration of fishes without damaging the target fish population or habitats.

## CONFLICT OF INTEREST

There are no conflict of interests to declare.

## AUTHOR CONTRIBUTIONS

A.M., H.Y. and A.I. conceived the ideas; A.M. and K.S. designed the field survey. K.S. collected and analyzed the data under supervision by A.M., K.W. and H.Y. A.M. led the writing of the manuscript. All the authors contributed critically to the drafts and approved the submission.

## DATA ACCESSIBILITY

Data available from the Dryad Digital Repository: https://doi.org/10.5061/dryad.5hb0fb8.

## References

[ece34653-bib-0001] Barnes, M. A. , & Turner, C. R. (2016). The ecology of environmental DNA and implications for conservation genetics. Conservation Genetics, 17, 1–17. 10.1007/s10592-015-0775-4.

[ece34653-bib-0002] Barnes, M. , Turner, C. R. , Jerde, C. L. , Renshaw, M. A. , Chadderton, W. L. , & Lodge, D. M. (2014). Environmental conditions influence eDNA persistence in aquatic systems. Environmental Science and Technology, 48, 1819–1827. 10.1021/es404734p. 24422450

[ece34653-bib-0003] Biggs, J. , Ewald, N. , Valentini, A. , Gaboriaud, C. , Dejean, T. , Griffiths, R. A. , & Dunn, F. (2015). Using eDNA to develop a national citizen science‐based monitoring programme for the great crested newt (*Triturus cristatus*). Biological Conservation, 183, 19–28. 10.1016/j.biocon.2014.11.029.

[ece34653-bib-0004] Chambert, T. , Pilliod, D. S. , Goldberg, C. S. , & Takahara, T. (2018). An analytical framework for estimating aquatic species density from environmental DNA. Ecology and Evolution, 10.1002/ece3.3764. PMC5869225 29607039

[ece34653-bib-0005] Darling, J. A. , & Mahon, A. R. (2011). From molecules to management: Adopting DNA‐based methods for monitoring biological invasions in aquatic environments. Environmental Research, 111, 978–988. 10.1016/j.envres.2011.02.001. 21353670

[ece34653-bib-0006] Doi, H. , Inui, R. , Akamatsu, Y. , Kanno, K. , Yamanaka, H. , Takahara, T. , & Minamoto, T. (2016). Environmental DNA analysis for estimating the abundance and biomass of stream fish. Freshwater Biology, 62, 30–39. 10.1111/fwb.12846.

[ece34653-bib-0007] Dunker, K. J. , Sepulveda, A. J. , Massengill, R. L. , Olsen, J. B. , Russ, O. L. , Wenburg, J. K. , & Antonovich, A. (2016). Potential of environmental DNA to evaluate Northern pike (*Esox lucius*) eradication efforts: An experimental test and case study. PLoS ONE, 11, e0162277 10.1371/journal.pone.0162277 27626271 PMC5023132

[ece34653-bib-0008] Eichmiller, J. J. , Bajer, P. G. , & Sorensen, P. W. (2014). The relationship between the distribution of common carp and their environmental DNA in a small lake. PLoS ONE, 9, e112611 10.1371/journal.pone.0112611 25383965 PMC4226586

[ece34653-bib-0009] Erickson, R. A. , Rees, C. B. , Coulter, A. A. , Merkes, C. M. , McCalla, S. G. , Touzinsky, K. F. , … Amberg, J. J. (2016). Detecting the movement and spawning activity of bigheaded carps with environmental DNA. Molecular Ecology Resources, 16, 957–965. 10.1111/1755-0998.12533. 27087387 PMC6680351

[ece34653-bib-0010] Ficetola, G. F. , Miaud, C. , Pompanon, F. , & Taberlet, P. (2008). Species detection using environmental DNA from water samples. Biology Letters, 4, 423–425. 10.1098/rsbl.2008.0118. 18400683 PMC2610135

[ece34653-bib-0011] Ghosal, R. , Eichmiller, J. J. , Witthuhn, B. A. , & Sorensen, P. W. (2018). Attracting Common Carp to a bait site with food reveals strong positive relationships between fish density, feeding activity, environmental DNA, and sex pheromone release that could be used in invasive fish management. Ecology and Evolution, 8, 6714–6727. 10.1002/ece3.4169. 30220992 PMC6137546

[ece34653-bib-0012] Goldberg, C. S. , Pilliod, D. S. , Arkle, R. S. , & Waits, L. P. (2011). Molecular detection of vertebrates in stream water: A demonstration using Rocky Mountain tailed frogs and Idaho giant salamanders. PLoS ONE, 6, e22746 10.1371/journal.pone.0022746 21818382 PMC3144250

[ece34653-bib-0013] Imamura, A. (2014). A detailed assessment of the distribution of *Opsariichthys uncirostris uncirostris* along the shoreline of Lake Biwa. Japanese Journal of Conservation Ecology, 19, 151–158. 10.18960/hozen.19.2_151.

[ece34653-bib-0014] Jane, S. F. , Wilcox, T. M. , McKelvey, K. S. , Young, M. K. , Schwartz, M. K. , Lowe, W. H. , … Whiteley, A. R. (2015). Distance, flow and PCR inhibition: eDNA dynamics in two headwater streams. Molecular Ecology Resources, 15, 216–227. 10.1111/1755-0998.12285. 24890199

[ece34653-bib-0015] Japan Ministry of the Environment . (2010). Appendix of the red list revised edition: Brackish and freshwater fishes. Tokyo, Japan: Ministry of the Environment.

[ece34653-bib-0016] Jerde, C. L. , Mahon, A. R. , Chadderton, W. L. , & Lodge, D. M. (2011). "Sight‐unseen" detection of rare aquatic species using environmental DNA. Conservation Letters, 4, 150–157. 10.1111/j.1755-263X.2010.00158.x.

[ece34653-bib-0017] Klobucar, S. L. , Rodgers, T. W. , & Budy, P. (2017). At the forefront: Evidence of the applicability of using environmental DNA to quantify the abundance of fish populations in natural lentic waters with additional sampling considerations. Canadian Journal of Fisheries and Aquatic Sciences, 74, 2030–2034. 10.1139/cjfas-2017-0114.

[ece34653-bib-0018] Klymus, K. E. , Richter, C. A. , Chapman, D. C. , & Paukert, C. (2015). Quantification of eDNA shedding rates from invasive bighead carp *Hypophthalmichthys nobilis* and silver carp *Hypophthalmichthys molitrix* . Biological Conservation, 183, 77–84. 10.1016/j.biocon.2014.11.020.

[ece34653-bib-0019] Lacoursière‐Roussel, A. , Côté, G. , Leclerc, V. , & Bernatchez, L. (2016). Quantifying relative fish abundance with eDNA: A promising tool for fisheries management. Journal of Applied Ecology, 53, 1148–1157. 10.1111/1365-2664.12598.

[ece34653-bib-0020] Laramie, M. B. , Pilliod, D. S. , & Goldberg, C. S. (2014). Characterizing the distribution of an endangered salmonid using environmental DNA analysis. Biological Conservation, 183, 29–37. 10.1016/j.biocon.2014.11.025.

[ece34653-bib-0021] Lodge, D. M. , Turner, C. R. , Jerde, C. L. , Barnes, M. A. , Chadderton, L. , Egan, S. P. , … Pfrender, M. E. (2012). Conservation in a cup of water: Estimating biodiversity and population abundance from environmental DNA. Molecular Ecology, 21, 2555–2558. 10.1111/j.1365-294X.2012.05600.x. 22624944 PMC3412215

[ece34653-bib-0022] Maruyama, A. , Nakamura, K. , Yamanaka, H. , Kondoh, M. , & Minamoto, T. (2014). The release rate of environmental DNA from juvenile and adult fish. PLoS ONE, 9, e114639 10.1371/journal.pone.0114639 25479160 PMC4257714

[ece34653-bib-0023] Minamoto, T. , Naka, T. , Moji, K. , & Maruyama, A. (2016). Techniques for the practical collection of environmental DNA: Filter selection, preservation, and extraction. Limnology, 17, 23–32. 10.1007/s10201-015-0457-4.

[ece34653-bib-0024] Miya, M. , Sato, Y. , Fukunaga, T. , Sado, T. , Poulsen, J. Y. , Sato, K. , … Iwasaki, W. (2015). MiFish, a set of universal PCR primers for metabarcoding environmental DNA from fishes: Detection of more than 230 subtropical marine species. Royal Society Open Science, 2, 150088 10.1098/rsos.150088. 26587265 PMC4632578

[ece34653-bib-0025] Mizumoto, H. , Urabe, H. , Kanbe, T. , Fukushima, M. , & Araki, H. (2017). Establishing an environmental DNA method to detect and estimate the biomass of Sakhalin taimen, a critically endangered Asian salmonid. Limnology, 19, 219–227. 10.1007/s10201-017-0535-x.

[ece34653-bib-0026] Nakagawa, H. , Yamamoto, S. , Sato, Y. , Sado, T. , Minamoto, T. , & Miya, M. (2018). Comparing local‐ and regional‐ scale estimations of the diversity of stream fish using eDNA metabarcoding and conventional observation methods. Freshwater Biology, 10.1111/fwb.13094.

[ece34653-bib-0027] Nakamura, M. (1951). The life history of a cyprinid fish, *Opsariichihys uncirosiris* (Temminck et Schlegel) in Lake Biwa (in Japanese). Miscellaneous Reports of the Research Institute for Natural Resources, 19–21, 70–78.

[ece34653-bib-0028] Nevers, M. B. , Byappanahalli, M. N. , Morris, C. C. , Shively, D. , Przybyla‐Kelly, K. , Spoljaric, A. M. , … Roseman, E. F. (2018). Environmental DNA (eDNA): A tool for quantifying the abundant but elusive round goby (*Neogobius melanostomus*). PLoS ONE, 13, e0191720 10.1371/journal.pone.0191720 29357382 PMC5777661

[ece34653-bib-0029] Pilliod, D. S. , Goldberg, C. S. , Arkle, R. S. , & Waits, L. P. (2014). Factors influencing detection of eDNA from a stream‐dwelling amphibian. Molecular Ecology Resources, 14, 109–116. 10.1111/1755-0998.12159. 24034561

[ece34653-bib-0030] Pilliod, D. S. , Goldberg, C. S. , Arkle, R. S. , Waits, L. P. , & Richardson, J. (2013). Estimating occupancy and abundance of stream amphibians using environmental DNA from filtered water samples. Canadian Journal of Fisheries and Aquatic Sciences, 70, 1123–1130. 10.1139/cjfas-2013-0047.

[ece34653-bib-0031] R Core Team . (2015). R: A language and environment for statistical computing. Vienna, Austria: R Foundation for Statistical Computing.

[ece34653-bib-0032] Rice, C. J. , Larson, E. R. , & Taylor, C. A. (2018). Environmental DNA detects a rare large river crayfish but with little relation to local abundance. Freshwater Biology, 63, 443–455. 10.1111/fwb.13081.

[ece34653-bib-0033] Seymour, M. , Durance, I. , Cosby, B. J. , Ransom‐Jones, E. , Deiner, K. , Ormerod, S. J. , … Creer, S. (2018). Acidity promotes degradation of multi‐species environmental DNA in lotic mesocosms. Communications Biology, 1, 4 10.1038/s42003-017-0005-3. 30271891 PMC6123786

[ece34653-bib-0034] Shogren, A. J. , Tank, J. L. , Andruszkiewicz, E. A. , Olds, B. , Jerde, C. , & Bolster, D. (2016). Modelling the transport of environmental DNA through a porous substrate using continuous flow‐through column experiments. Journal of the Royal Society Interface, 13, 20160290 10.1098/rsif.2016.0290. PMC4938091 27251680

[ece34653-bib-0035] Shogren, A. J. , Tank, J. L. , Andruszkiewicz, E. , Olds, B. , Mahon, A. R. , Jerde, C. L. , & Bolster, D. (2017). Controls on eDNA movement in streams: Transport, retention, and resuspension. Scientific Reports, 7, 5065 10.1038/s41598-017-05223-1. 28698557 PMC5506058

[ece34653-bib-0036] Spear, S. F. , Groves, J. D. , Williams, L. A. , & Waits, L. P. (2015). Using environmental DNA methods to improve detectability in a hellbender (*Cryptobranchus alleganiensis*) monitoring program. Biological Conservation, 183, 38–45. 10.1016/j.biocon.2014.11.016.

[ece34653-bib-0037] Strickler, K. M. , Fremier, A. K. , & Goldberg, C. S. (2015). Quantifying effects of UV‐B, temperature, and pH on eDNA degradation in aquatic microcosms. Biological Conservation, 183, 85–92. 10.1016/j.biocon.2014.11.038.

[ece34653-bib-0038] Sutherland, W. J. (1998). The importance of behavioural studies in conservation biology. Animal Behaviour, 56, 801–809. 10.1006/anbe.1998.0896. 9790690

[ece34653-bib-0039] Takahara, T. , Minamoto, T. , Yamanaka, H. , Doi, H. , & Kawabata, Z. I. (2012). Estimation of fish biomass using environmental DNA. PLoS ONE, 7, e35868 10.1371/journal.pone.0035868 22563411 PMC3338542

[ece34653-bib-0040] Tanaka, S. (1970). Studies on the growth of “hasu”, *Opasariichthys uncirostaris*, in Lake Biwa I. On the body length at each age and the growth curve estimated from the spawning populations. Japanese Journal of Ecology, 20, 13–25.

[ece34653-bib-0041] Thomsen, P. , Kielgast, J. O. S. , Iversen, L. L. , Wiuf, C. , Rasmussen, M. , Gilbert, M. T. P. , … Willerslev, E. (2012). Monitoring endangered freshwater biodiversity using environmental DNA. Molecular Ecology, 21, 2565–2573. 10.1111/j.1365-294X.2011.05418.x. 22151771

[ece34653-bib-0042] Tsuji, S. , Ushio, M. , Sakurai, S. , Minamoto, T. , & Yamanaka, H. (2017). Water temperature‐dependent degradation of environmental DNA and its relation to bacterial abundance. PLoS ONE, 12, e0176608. 28448613 10.1371/journal.pone.0176608 PMC5407774

[ece34653-bib-0043] Tsuji, S. , Yamanaka, H. , & Minamoto, T. (2017). Effects of water pH and proteinase K treatment on the yield of environmental DNA from water samples. Limnology, 18, 1–7. 10.1007/s10201-016-0483-x.

[ece34653-bib-0044] Tsunoda, H. , Urano, T. , & Ohira, M. (2015). Comparison of food habits between native Amur three‐lips (*Opsariichthys uncirostris uncirostris*) and non‐native largemouth bass (*Micropterus salmoides*) in Lake Biwa, Japan. Annales De Limnologie‐International Journal of Limnology, 51, 273–280. 10.1051/limn/2015021.

[ece34653-bib-0045] Wilcox, T. M. , McKelvey, K. S. , Young, M. K. , Sepulveda, A. J. , Shepard, B. B. , Jane, S. F. , … Schwartz, M. K. (2016). Understanding environmental DNA detection probabilities: A case study using a stream‐dwelling char *Salvelinus fontinalis* . Biological Conservation, 194, 209–216. 10.1016/j.biocon.2015.12.023.

[ece34653-bib-0046] Yamamoto, S. , Minami, K. , Fukaya, K. , Takahashi, K. , Sawada, H. , Murakami, H. , … Kondo, M. (2016). Environmental DNA as a ‘snapshot’ of fish distribution: A case study of Japanese Jack Mackerel in Maizuru Bay. Sea of Japan. Plos ONE, 11, e0149786. 26933889 10.1371/journal.pone.0149786 PMC4775019

[ece34653-bib-0047] Yamanaka, H. , & Minamoto, T. (2016). The use of environmental DNA of fishes as an efficient method of determining habitat connectivity. Ecological Indicators, 62, 147–153. 10.1016/j.ecolind.2015.11.022.

[ece34653-bib-0048] Yamanaka, H. , Takao, D. , Maruyama, A. , & Imamura, A. (2018). Species‐specific detection of the endangered piscivorous cyprinid fish *Opsariichthys uncirostris uncirostris*, three‐lips, using environmental DNA analysis. Ecological Research., 33(5), 1075–1078. 10.1007/s11284-018-1612-2.

